# Supplementary figures and images for: Escherichia coli transcription factors of unknown function: sequence features and possible evolutionary relationships
Source: PeerJ. 2022 Jul 20;10:e13772. doi: 10.7717/peerj.13772 (PMC9308461; doi:10.7717/peerj.13772)

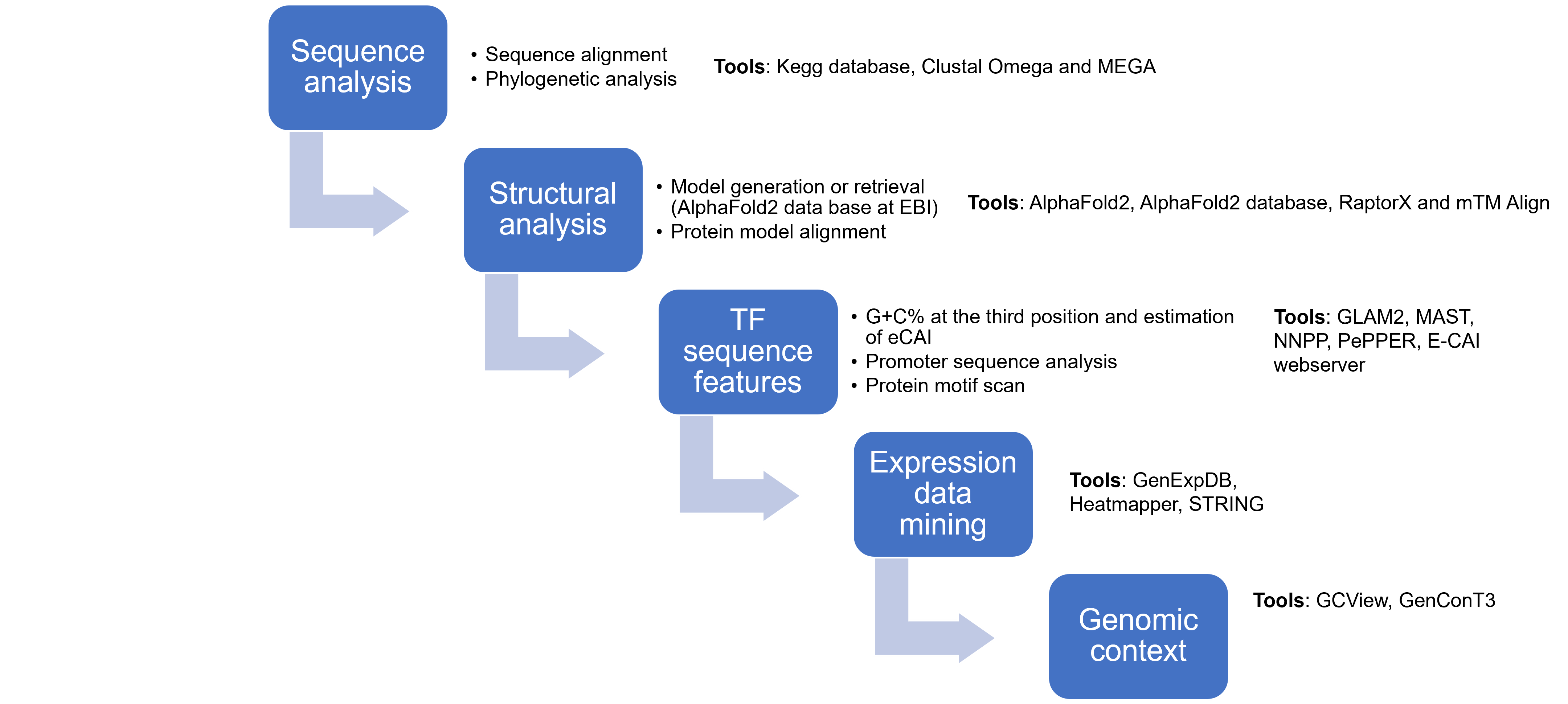

Supplement: Supplemental Information 1 — Aspects to be explored in the sequence and structural features of TFs of unknown function are indicated and the tools used. [file peerj-10-13772-s001.png]

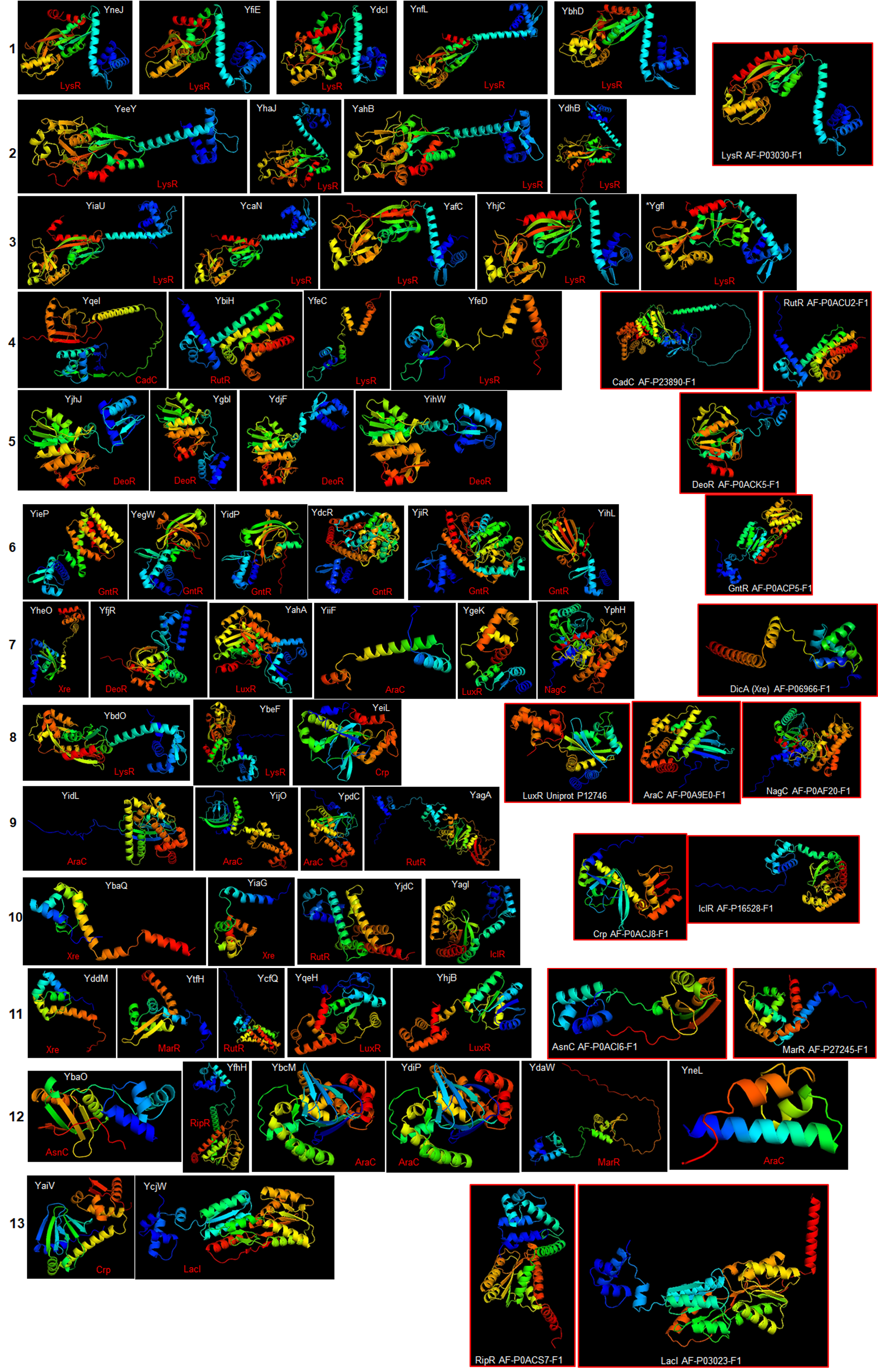

Supplement: Supplemental Information 2 — Each model is shown by group and in the approximate position shown in the structural alignments. The blue color indicates the N-terminal end, and the red indicates the C-terminal end. For groups 7 and 8, proteins are shown in the position for the overall alignment (Figure 2). The predicted family for each TF is indicated in red, and models with red frames indicate the reference structure for the representative member of the stated family of TF. [file peerj-10-13772-s002.png]

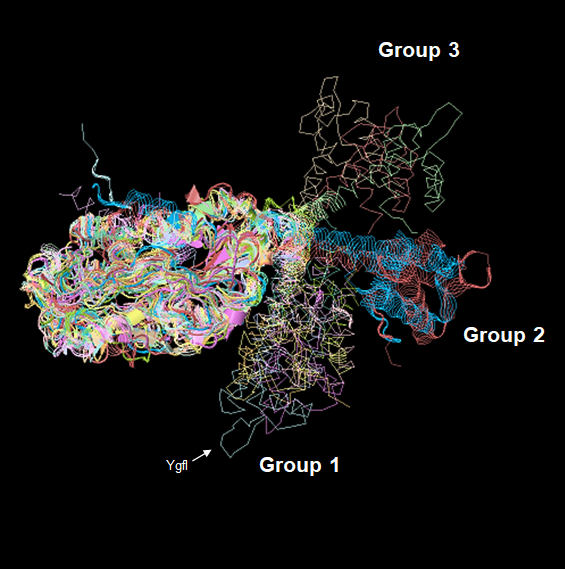

Supplement: Supplemental Information 3 — The relative position of each group is in relation to the most distant set of the phylogenetic comparison. YgfL is structurally similar to Group 1 in Figure 2, even though the sequence is more divergent. [file peerj-10-13772-s003.png]

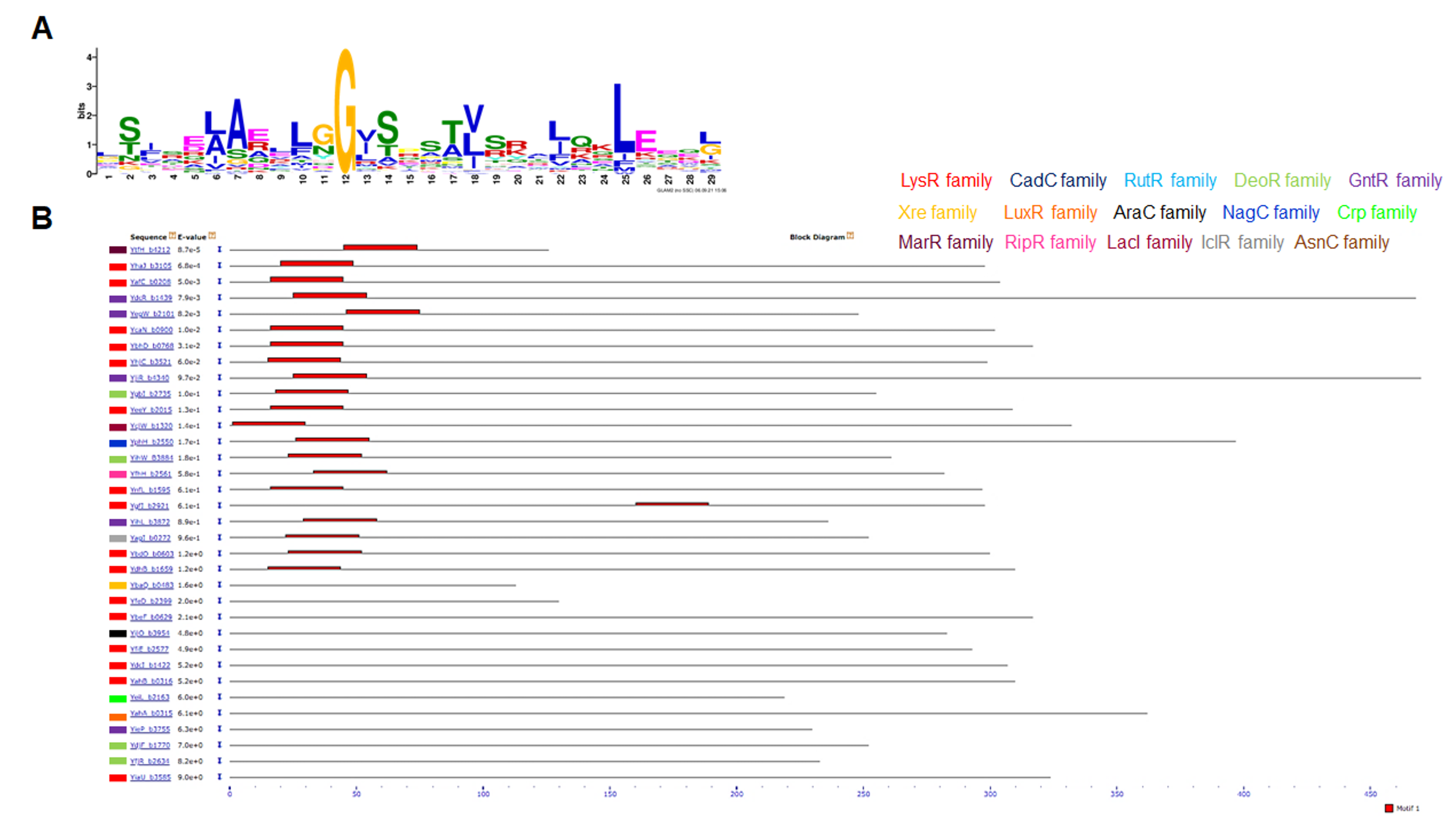

Supplement: Supplemental Information 4 — Using GLAM2 (Frith et al., 2008) a motif was found with the following regular expression: L[ST][FI].E[AL]A[ER].LGG?[IV]SS[AT][LV]SR.[IL]?QKL?E..[GL] (Panel A). Then, using MAST (Bailey & Gribskov, 1998), the motif was scanned and mapped in the protein sequences, finding a positive consensus in the following sequences: YtfH (set 12-RipR), YhaJ (set 2-LysR), YafC (set 3-LysR), YdcR (set 6-GntR), YegW (set 6-GntR), YcaN (set 3-LysR), YbhD (set 1-LysR), YhjC (set 3-LysR), YjiR (set 6-DeoR), YgbI (set 5-DeoR), YeeY (set 2-LysR), YcjW (set 13-LacI), YphH (set 7-NagC), YihW (set 5-DeoR), YfhH (set 12-RipR), YnfL (set 1-LysR), YgfI (set 3–4-LysR), YihL (set 6-GntR), YagI, (set 10-IclR), YbdO (set 8-LysR), YdhB (set 2-LysR). Low hit sequences were also indicated. On each hit using MAST, the family of each TF is indicated using the color code indicated on top of the MAST result. For comparison with the localization of the motif, Panel C provides the Prosite database scan (Sigrist et al., 2012) of each TF to locate the DNA binding domain or other well conserved domains. [file peerj-10-13772-s004.png]

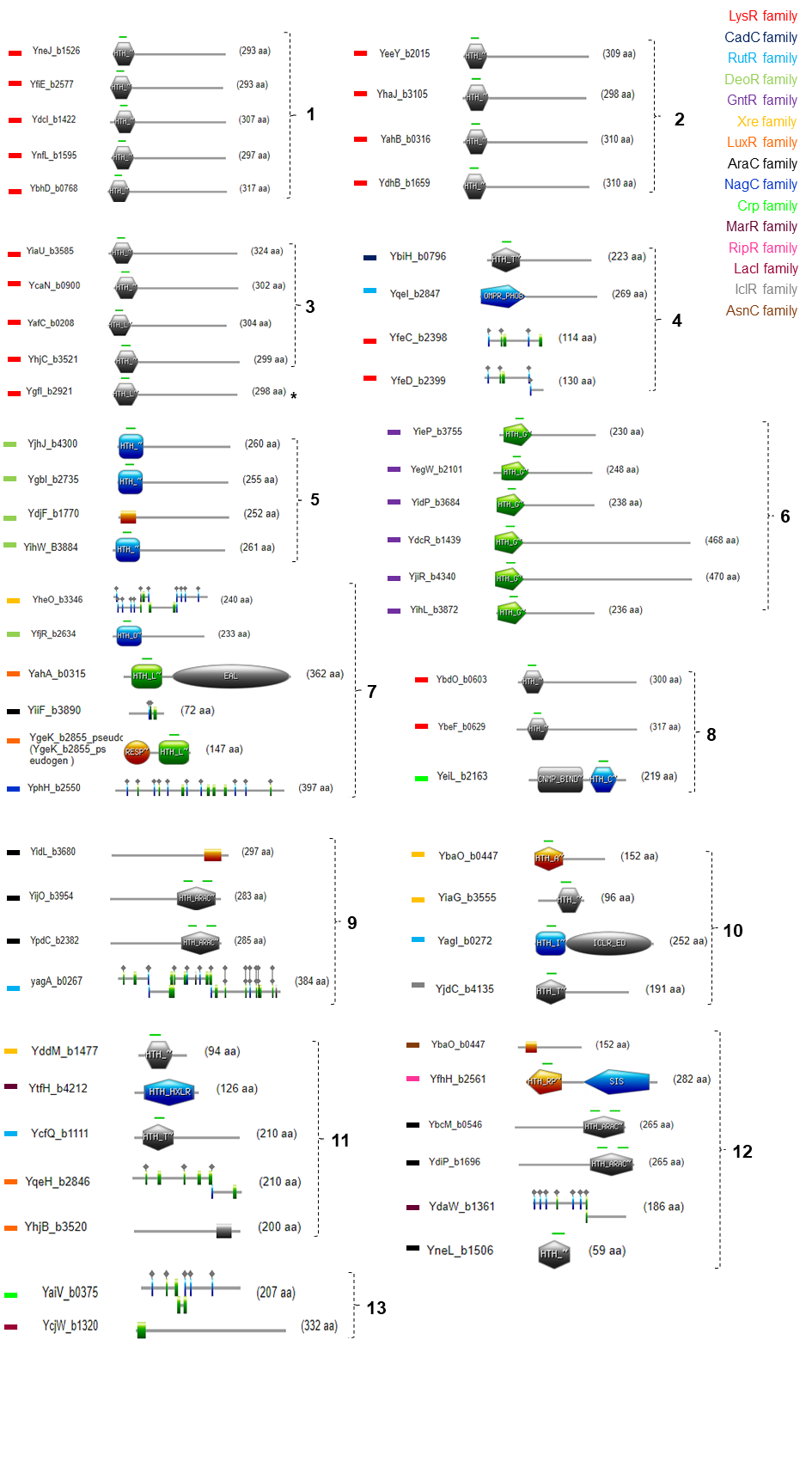

Supplement: Supplemental Information 5 — Using GLAM2 (Frith et al., 2008) a motif was found with the following regular expression: L[ST][FI].E[AL]A[ER].LGG?[IV]SS[AT][LV]SR.[IL]?QKL?E..[GL] (Panel A). Then, using MAST (Bailey & Gribskov, 1998), the motif was scanned and mapped in the protein sequences, finding a positive consensus in the following sequences: YtfH (set 12-RipR), YhaJ (set 2-LysR), YafC (set 3-LysR), YdcR (set 6-GntR), YegW (set 6-GntR), YcaN (set 3-LysR), YbhD (set 1-LysR), YhjC (set 3-LysR), YjiR (set 6-DeoR), YgbI (set 5-DeoR), YeeY (set 2-LysR), YcjW (set 13-LacI), YphH (set 7-NagC), YihW (set 5-DeoR), YfhH (set 12-RipR), YnfL (set 1-LysR), YgfI (set 3–4-LysR), YihL (set 6-GntR), YagI, (set 10-IclR), YbdO (set 8-LysR), YdhB (set 2-LysR). Low hit sequences were also indicated. On each hit using MAST, the family of each TF is indicated using the color code indicated on top of the MAST result. For comparison with the localization of the motif, Panel C provides the Prosite database scan (Sigrist et al., 2012) of each TF to locate the DNA binding domain or other well conserved domains. [file peerj-10-13772-s005.png]

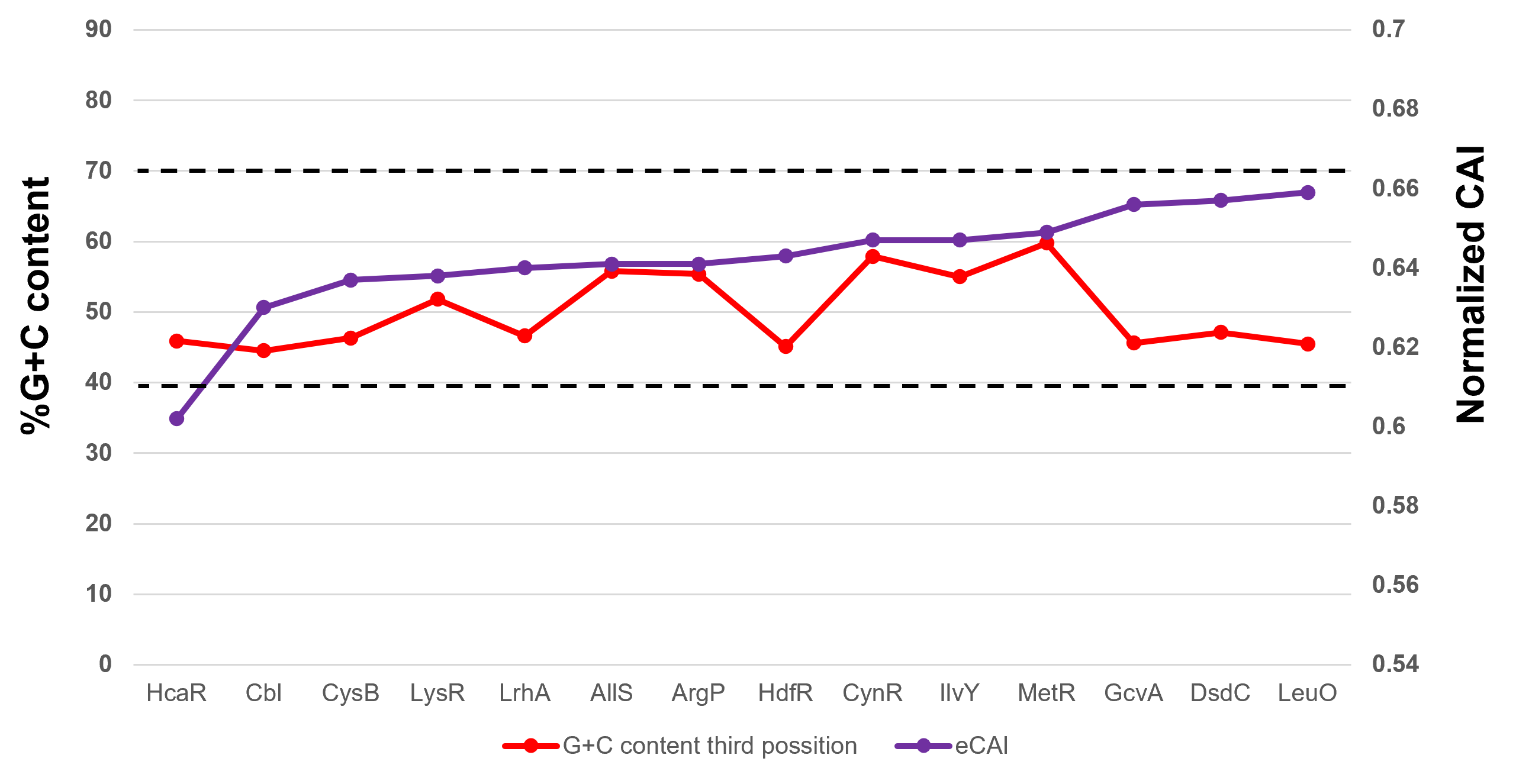

Supplement: Supplemental Information 6 — Here, 15 experimentally validated LysR family TFs were analyzed for the normalized CAI, ordered from the lowest to the highest value (purple data points), and then plotted along with the %G+C content (red data points), indicating each TF. Horizontal dashed lines were used to indicate the limits of normalized CAI values for annotated and functional TFs to facilitate comparison [file peerj-10-13772-s006.png]

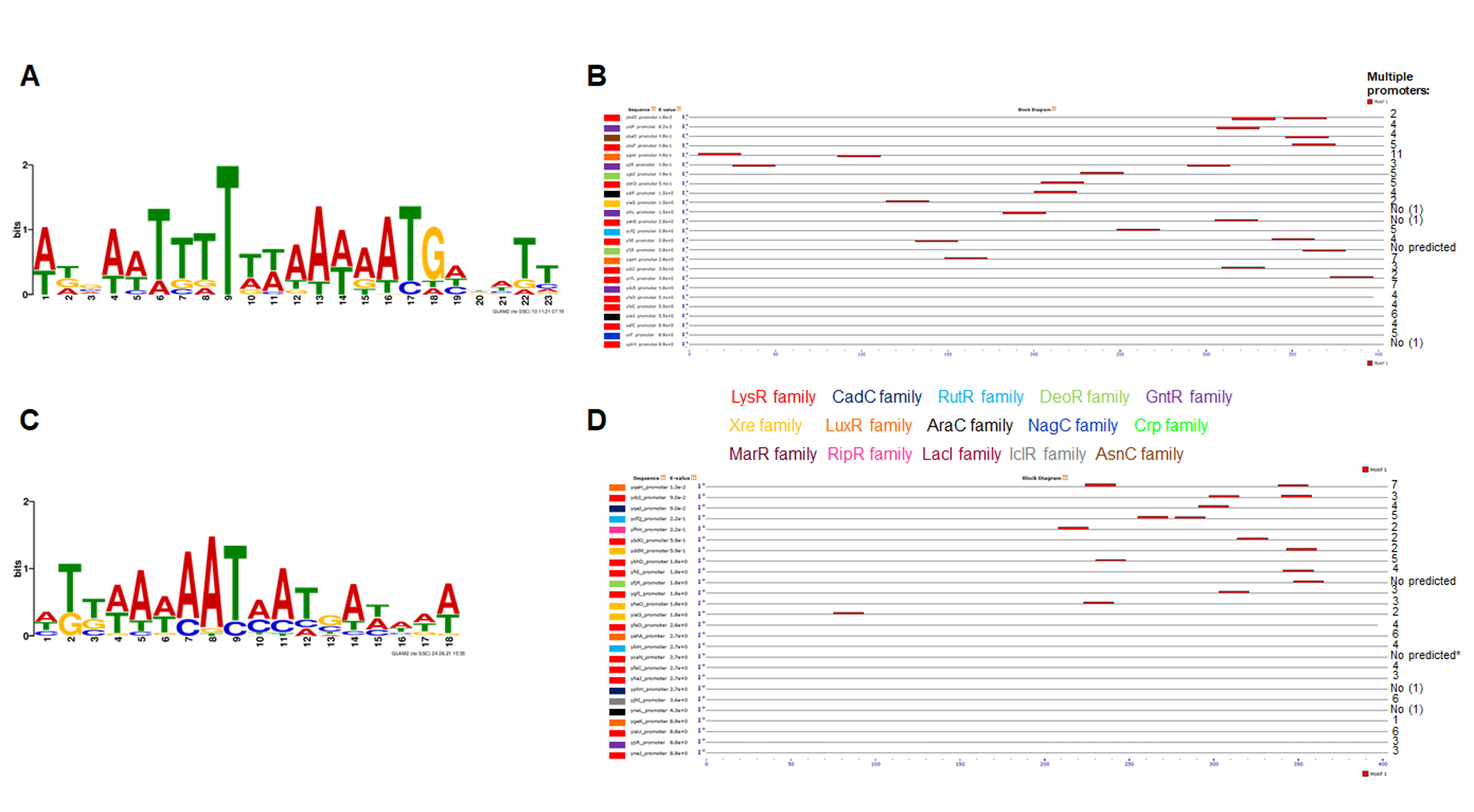

Supplement: Supplemental Information 7 — In Panel A and C, Weblogo analysis derived from GLAM2 analysis of 400 bp and the ATG sequence for each TF of unknown function For panel A, the regular expression is [AT][ATG][ACG][AT][AT]TTTT.{0,75}[AT][AT]AA[AT][AG]ATG[ACT][ATG][AT]TT and for Panel B the regular expression is [ACT][TG][TG][AT]A[AT][AC]AT?[AC]AT[CG]A[ACT][AT][AT][AT]. Each sequence was then analyzed using MAST for relative position and abundance. Panels B and C show the map, the number of motifs shown on Panels A and C, and the number of predicted promoter sequences described in the Methods section. Low match results were also included (absence of red boxes). The family for each TF is indicated in the color code between panels B and D. Promoter prediction was conducted with NNPP, except for those sequences where no predicted result was found, was further analyzed with PePPER. [file peerj-10-13772-s007.png]

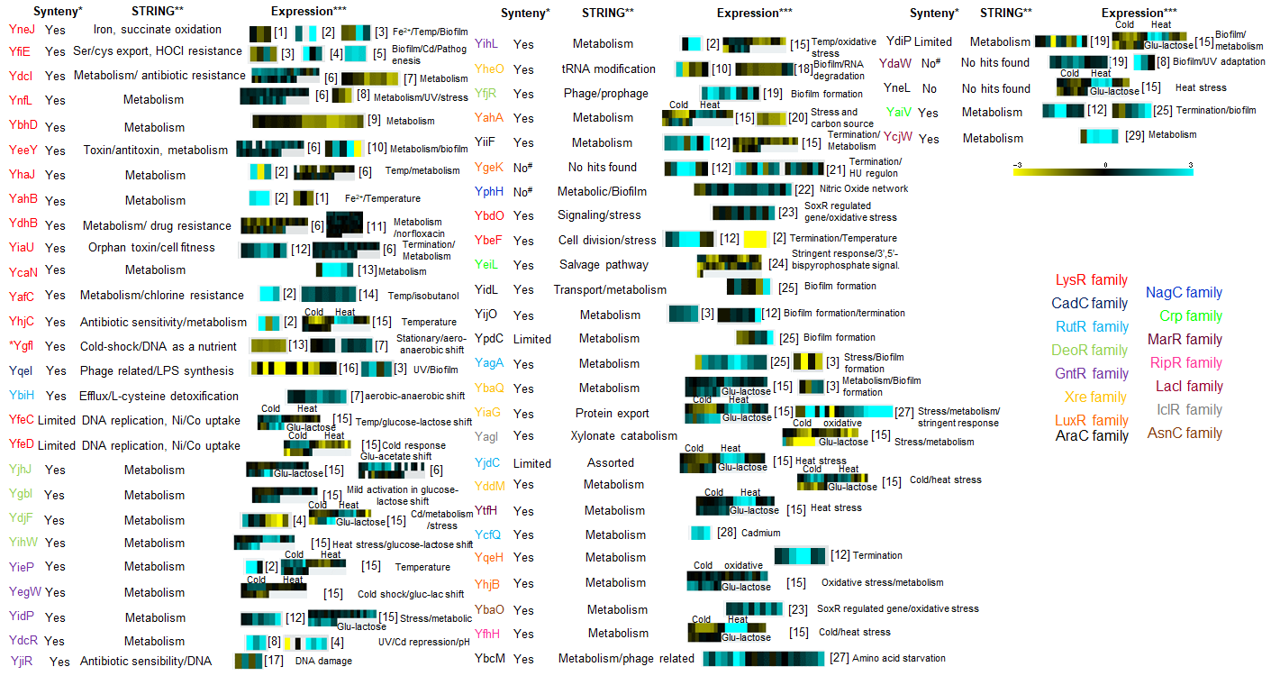

Supplement: Supplemental Information 8 — The data presented is Synteny (using GeConT 3), putative interacting partners that may represent a function by STRING analysis and expression data from https://genexpdb.okstate.edu/databases/genexpdb/. Legend: Synteny* Presence of other arrangements in diverse bacteria. # Indicates that different arrangements are found in E. coli strains, although no synteny outside E. coli is found. STRING** Predicted functional partners or the relevant function of partners. Expression*** snapshot of data heatmap of representative experiments either relevant to STRING results or showing consistent regulation, and threshold expression are indicated in the far right of the figure. The reference for each experiment is shown here is indicated in brackets. On top of some datasets is displayed the corresponding condition that either expression or repression is observed. Data from different studies cannot be compared quantitatively, only qualitatively. All data details and expression profiles are presented in Files S2 and S3. [file peerj-10-13772-s008.png]

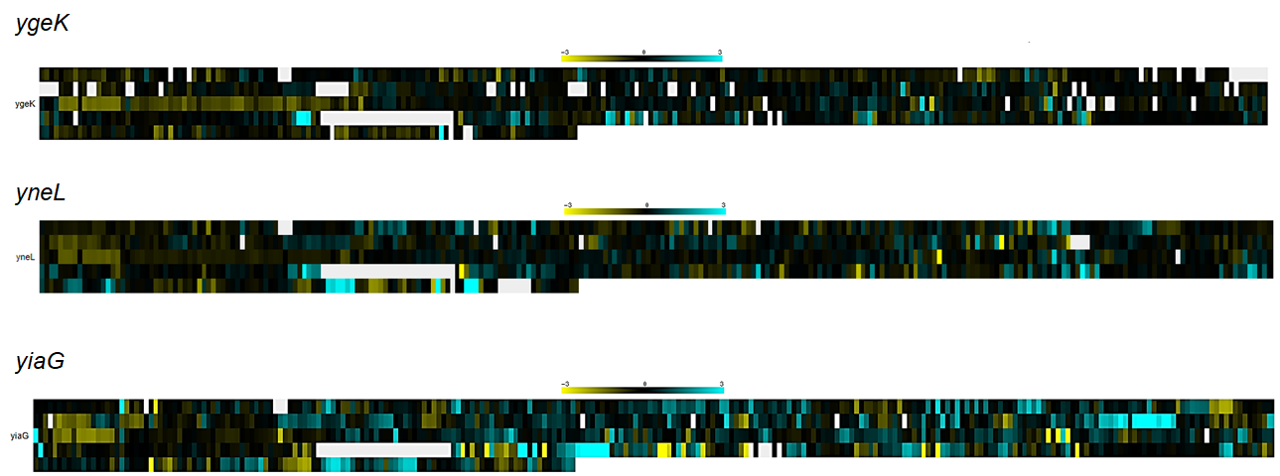

Supplement: Supplemental Information 9 — Two (YgeK and YneL) pseudogenes and a highly regulated TF (YiaG) are shown. Data retrieved from GenExpDB (https://genexpdb.okstate.edu/databases/genexpdb/). Heat map corresponds to −3 to 3 thresholds. [file peerj-10-13772-s009.png]

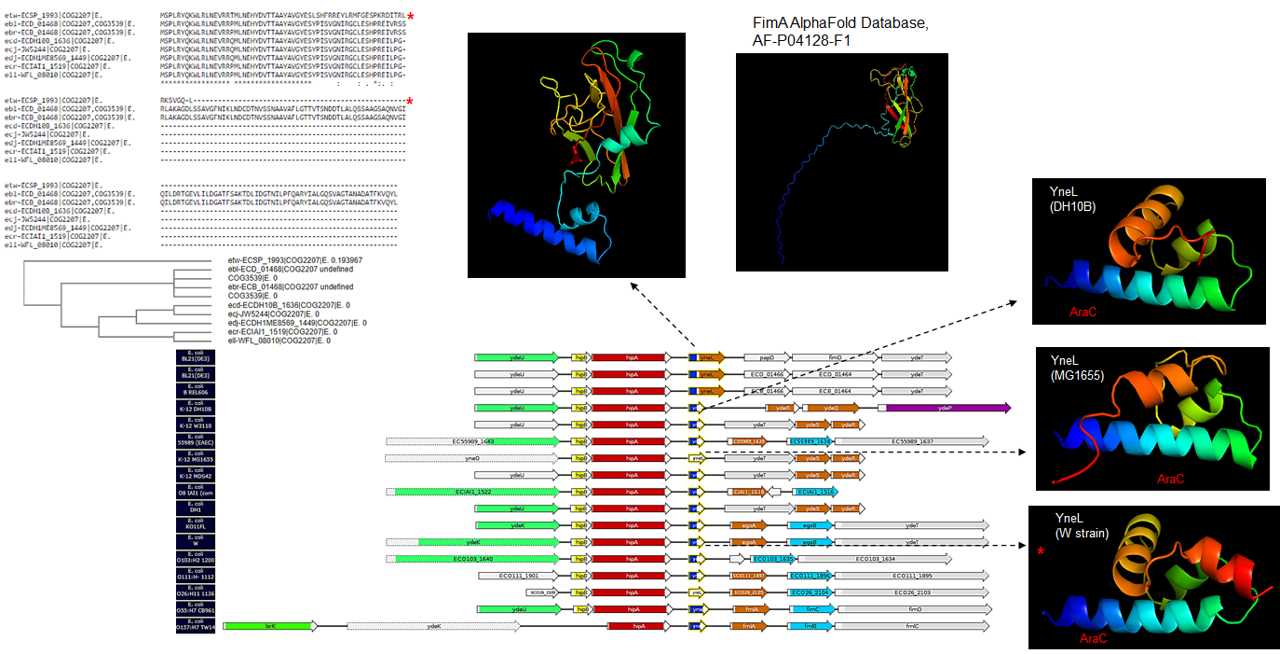

Supplement: Supplemental Information 10 — Sequence comparison was conducted with Clustal Omega, and synteny was determined with GeConT 3 tools. Dashed arrows indicate models of each case. The blue color indicates the N-terminal end, and the red indicates the C-terminal end. Synteny legend: blue box indicates AraC-type DNA-binding domain. The Orange box indicates P-pilus assembly protein, pilin FimA. FimA AlphaFold model (Accession number AF-P04128-F1) is shown. [file peerj-10-13772-s010.png]

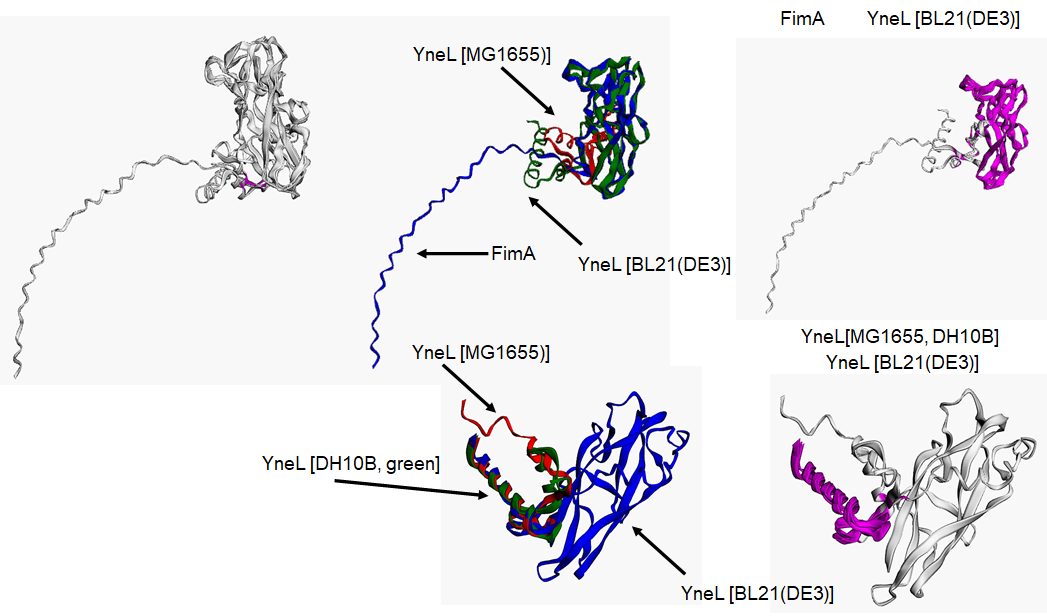

Supplement: Supplemental Information 11 — Color code indicates the common overlap between structures, and magenta indicates the common core in each comparison. Strong sequence and structural conservation are found between FimA and YneL from BL21 strain. [file peerj-10-13772-s011.png]

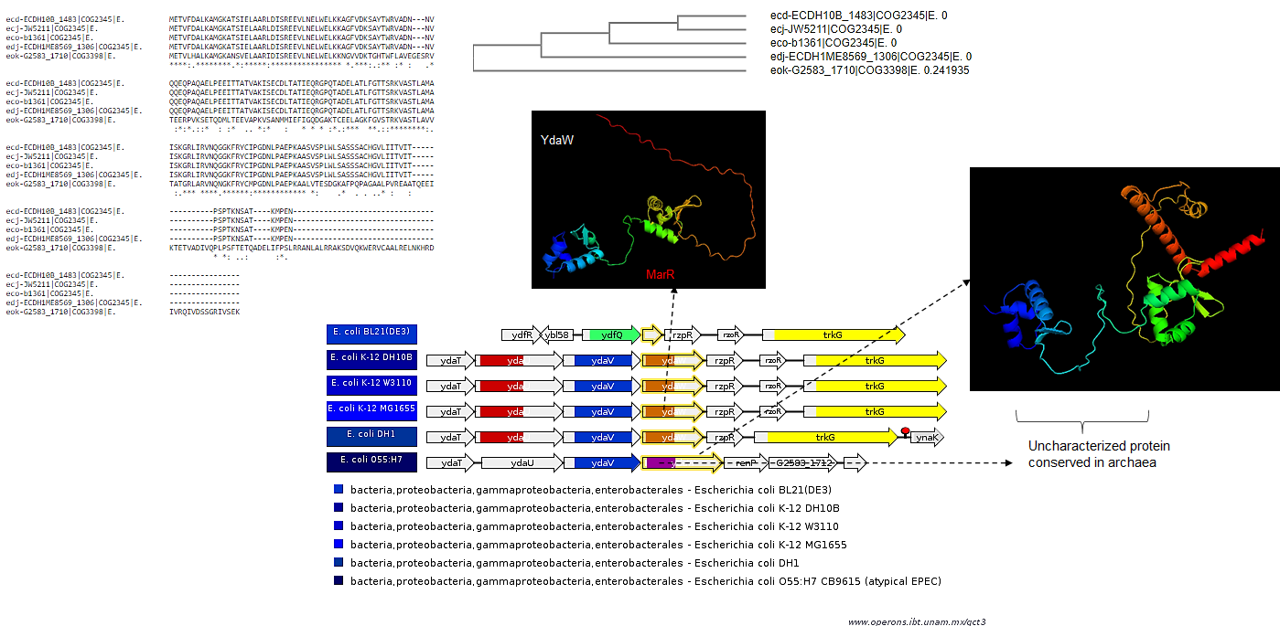

Supplement: Supplemental Information 12 — In strain O55:H7 a larger protein with a different structure is found. Orange box, MarR-type of DNA-binding protein. Light purple box indicates a sequence of an uncharacterized protein conserved in archaea, corresponding to the putative DNA-binding domain in YdaW. [file peerj-10-13772-s012.png]

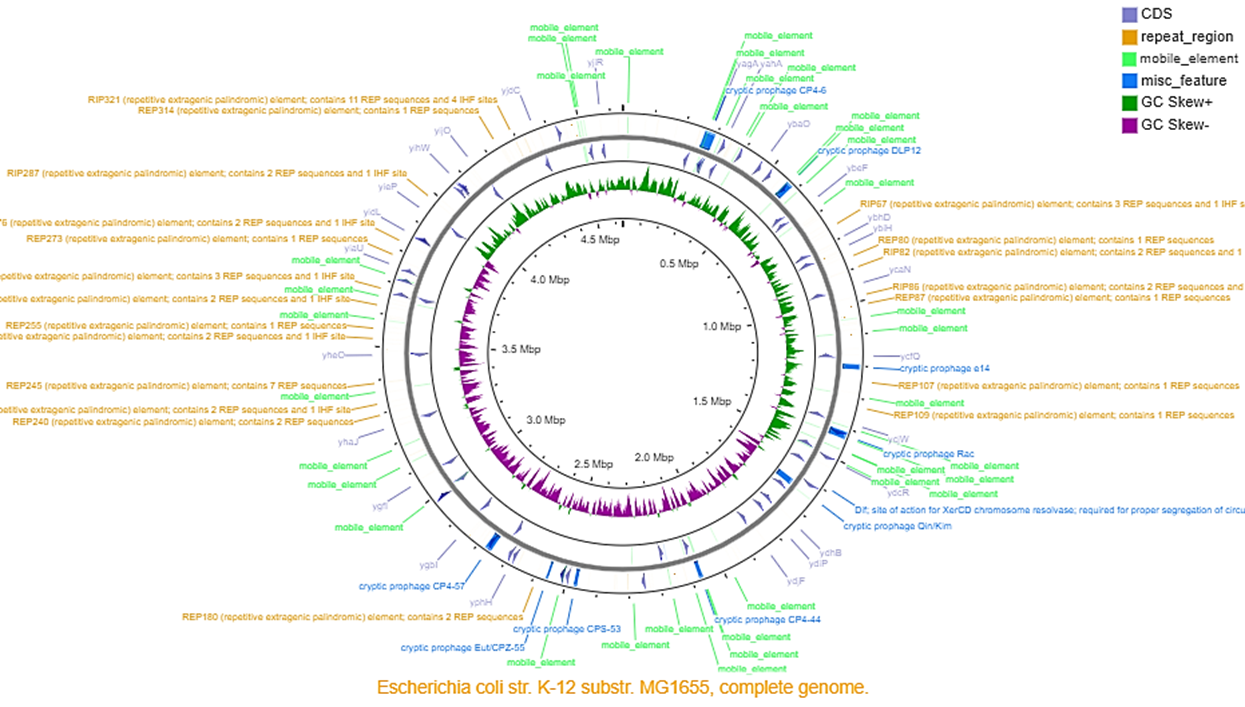

Supplement: Supplemental Information 13 — Genomic map for all 58 TFs showing simultaneously all the elements that suggest HGT: mobile elements, prophage elements, and repeated sequences. [file peerj-10-13772-s013.png]

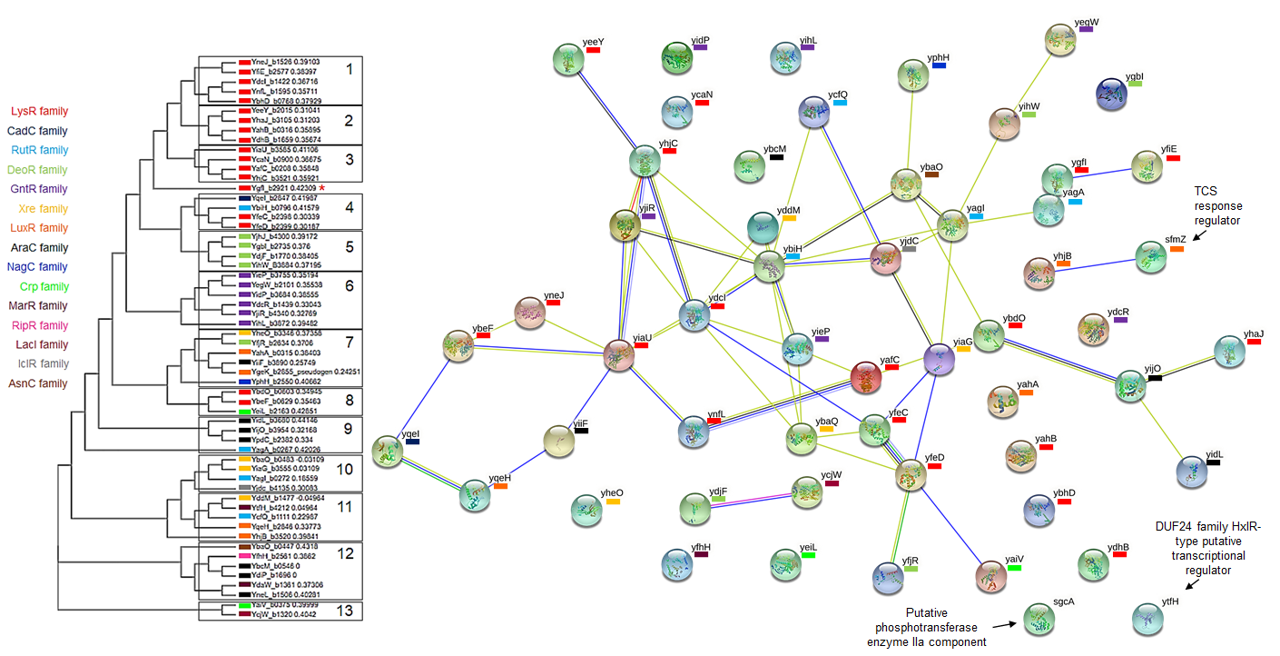

Supplement: Supplemental Information 14 — Each TF is manually indicated the family it belongs; for reference, on the left is shown the phylogenetic comparison shown in Figure 2. Additional regulators are shown with black arrows with its description. [file peerj-10-13772-s014.png]
