# Supplementary material for: Escherichia coli transcription factors of unknown function: sequence features and possible evolutionary relationships
Source: PeerJ. 2022 Jul 20;10:e13772. doi: 10.7717/peerj.13772 (PMC9308461; doi:10.7717/peerj.13772)
Supplement: Supplemental Information 15 — Predictions were conducted with NNPP or PePPER. [file peerj-10-13772-s015.docx]

**Promoter predictions for 58 prokaryotic sequences with score cutoff 0.80 (transcription start shown in larger font):**

**Promoter predictions for yafC_promoter :**

**Start End Score Promoter Sequence**
 148 193 0.94 TGTTTTTTCGCACTTTTGACTTTGAAACCTCTATTACGTCAACCCTTCCG
 301 346 0.89 CATTATGGAGAACATTAAATCGCATGACATATCACGCATCCTGATGGTTT
 319 364 0.88 ATCGCATGACATATCACGCATCCTGATGGTTTAATGACAAATAAATTCAA
 342 387 0.91 TGATGGTTTAATGACAAATAAATTCAAAATATCTCTTCTATTTTGCAACA

**Promoter predictions for yagA_promoter :**

**Start End Score Promoter Sequence**
 113 158 0.93 TCCTTGCTGAATCATTTTGTTCTACATTATAGAACAGCGTTCGTTTATTT

**Promoter predictions for yagI_promoter :**

**Start End Score Promoter Sequence**
 332 377 0.97 ATTTCATTGATTTTTCATCCCGAAAAAGGTACGTTTTCGCCTTAATTCCA

**Promoter predictions for yahA_promoter :**

**Start End Score Promoter Sequence**
 33 78 0.95 TTTTCAGTTGATCTGGATTGTTAAATTCATATAATGCGCCTTTGCTCATG
 78 123 0.95 TCATGAATGGATGCCAGTATGTAGTGGGAAATTATAAATATTGAAATAGT
 112 157 0.91 TAAATATTGAAATAGTCCAACTACTTCTTTATTACCAAAAATGAGTATCT
 158 203 0.99 ATCTGAATTTTAATATTGCATTCTTGCGTGATTATCTCCTGAGTTTGACT
 202 247 0.81 TTGACTTGTGATTACCTTTTTAAGGTATTTAGCGTAACTGTTTTTGAGCG
 238 283 0.99 ACTGTTTTTGAGCGAGCATCAGAGGTAAAGATAATCTTCTTGATAGTGAT

**Promoter predictions for yahB_promoter :**

**Start End Score Promoter Sequence**
 219 264 0.98 GGGTTTTCTGGCGGGTAACTGCGTGGGGGTAATTTCAGAATATGGTGGAC

**Promoter predictions for yaiV_promoter :**

**Start End Score Promoter Sequence**
 314 359 0.97 TATTCTTCACTCCGAAGAAATACTGGTAATTTAATCTAAATAATGCCCGT

**Promoter predictions for ybaO_promoter :**

**Start End Score Promoter Sequence**
 81 126 0.86 GAAATGTTAGTCAGCGTCGGTAGCGGATTTATTATGGGCAATGCGATGCC
 302 347 0.94 TCATTTTGTGCCGTGGTGTTTAAACCGCACAGAATAAATTGTCGTGATTT
 334 379 0.96 AATAAATTGTCGTGATTTCACCTTTAAAATAAAATTAAAAGAGAAAAAAA
 350 395 0.97 TTCACCTTTAAAATAAAATTAAAAGAGAAAAAAATTCTCTGTGGAAGGGC

**Promoter predictions for ybaQ_promoter :**

**Start End Score Promoter Sequence**
 317 362 1.00 TGGAGTTGGAGACTTGTTTAATGTGTTTGTATGATTCAGTATGTTCTTGC
 327 372 1.00 GACTTGTTTAATGTGTTTGTATGATTCAGTATGTTCTTGCATCGCTATTC

**Promoter predictions for ybcM_promoter :**

**Start End Score Promoter Sequence**
 94 139 0.99 GACTGTTGTTAATATTCCAGCAACAGTAACATATTTGCCCGTTGATGCAG
 174 219 0.87 GTGCTGTTCAAGGCCGAAATGATTTTGGCTATGCTGGGTTTGGTGGCGCA
 210 255 0.85 GGTTTGGTGGCGCATGTCCTCCTAAAGGAGATAAACCACATCATTACCAG
 317 362 0.92 GCGTTAGTTGGTTATATGCTTAATGCTAATAAAATCGCAACCGCTGAGAT

**Promoter predictions for ybdO_promoter :**

**Start End Score Promoter Sequence**
 142 187 0.90 GTGGGGTTGCCCGATCAGAAAACGCTTAATATCATTATGGGGAATAAATA
 355 400 0.99 GATTAAGTGAATATATCATGGAAGAAAAATATAACCGGAGTAGTGTATG

**Promoter predictions for ybeF_promoter :**

**Start End Score Promoter Sequence**
 38 83 0.96 TCACAATGGAAACCCGAAGCGACGACTAATAATATTGCTCCACGTTTACT
 81 126 0.92 GTTTACTGGAAAATATTTTAGCGCTACTAAACAATCCGGACTTCGAATAT
 195 240 0.97 TAATGCTTTACATCTCCTTCGCGTTTGTATATTTTCTTTATCACTTGCAG
 279 324 0.95 CCATTGATTAACCTATTGAAATAGTTGATTATGGTGAGCAAAAGCGTTCC
 354 399 1.00 TAATTTTTTAAATATTATTTTTCCATGAATAAAATTGGAGTAAGTGCGTG

**Promoter predictions for ybhD_promoter :**

**Start End Score Promoter Sequence**
 72 117 0.80 CCGTCAATTTCCAGATCGTTACCGGAACCCATAATTGCCATCAATATTTT
 205 250 0.99 GTATTTTTTTCATAATAATGCCCTCAATTTAAAAACATAACTTATTGCGA
 226 271 1.00 CCTCAATTTAAAAACATAACTTATTGCGATATGGTTACATTAAGGGCAAA
 252 297 0.90 CGATATGGTTACATTAAGGGCAAAGCATCTCTAATGACTTTAGCTCAACG
 278 323 0.92 ATCTCTAATGACTTTAGCTCAACGATTAATAACATTTTTTCATTAATTTA

**Promoter predictions for ybiH_promoter :**

**Start End Score Promoter Sequence**
 202 247 0.84 GGTGTAGTTTTCAGGGAGATACTGAAAAGGAAAATGACAAAAACCACAAT
 217 262 0.91 GAGATACTGAAAAGGAAAATGACAAAAACCACAATGTCATGAGGGAGCGG
 305 350 0.90 CTGTGACTGGCGTTGCTAAAATTCTTAGCCATACTGGACAACTCCCCTAT
 333 378 0.91 CCATACTGGACAACTCCCCTATGGAGTCATAATCTTAATCAATCATTTGA

**Promoter predictions for ycaN_promoter :**

**Start End Score Promoter Sequence**

**Promoter predictions for ycfQ_promoter :**

**Start End Score Promoter Sequence**
 223 268 0.88 GATAGATGTGATCTGGATCACATACAAGATATTTTTTATAACAATCATTA
 252 297 1.00 TATTTTTTATAACAATCATTAATTAATTTAAAAATCTTTAAATTTCAGTT
 271 316 1.00 TAATTAATTTAAAAATCTTTAAATTTCAGTTAAATAGAAAGCAATTATTT
 287 332 0.96 CTTTAAATTTCAGTTAAATAGAAAGCAATTATTTTTATGGGATCTGGACT
 324 369 0.95 TGGGATCTGGACTGGTGAACGGAGGTGATTATTTTTATAATAACCTTTCA

**Promoter predictions for ycjQ_promoter :**

**Start End Score Promoter Sequence**
 331 376 0.97 CCCGTGTTGTCATCGACCGGCGAACTCCATAAGATGTGCAGCATTCACGT

**Promoter predictions for ydaW_promoter :**

**Start End Score Promoter Sequence**

**Promoter predictions for ydcI_promoter :**

**Start End Score Promoter Sequence**
 128 173 0.85 TGACATTGCCTGCGAAAACTGTTCCCGAATCTCATCCGCCGTGATGCTGT
 246 291 0.94 AGAATTTACAAACTGTGATCTCGCCGCGAAAACATCAATATTATCCATTT
 315 360 0.99 TAATTGTTAATAATATTTTGCAATCAAGTTATCATAATCAAACAACTTCA

**Promoter predictions for ydcR_promoter :**

**Start End Score Promoter Sequence**
 24 69 0.89 AAGATGCTTTACTGACCGCATTTGATTTTTATTTTGAAGATAACGAGCTT
 52 97 0.99 TTATTTTGAAGATAACGAGCTTATCCCTTTACCTTCGCCATTAAATAGTC
 91 136 0.84 ATTAAATAGTCACGATCACTTTATTGAAGTACCTTTGAGCGTCGCCTCTA
 289 334 0.91 GCTTGGCAAAGAGTTATCGCTGGTGATGGTTTAATTACAGTTAACGAAAA
 333 378 0.88 CGAAAAGTTGTCATTTTTAACAACTGATATAGACTGCCGAATCATCTGCA
 343 388 1.00 TCATTTTTAACAACTGATATAGACTGCCGAATCATCTGCACATAATTACG
 354 399 0.98 AACTGATATAGACTGCCGAATCATCTGCACATAATTACGATTCGATAATG

**Promoter predictions for yddM_promoter :**

**Start End Score Promoter Sequence**
 321 366 0.83 AAATGGATTATTGTTGCGTAGGAAGGAACAATAATGATAGTGTTCCCAGT
 329 374 0.98 TATTGTTGCGTAGGAAGGAACAATAATGATAGTGTTCCCAGTGAAGGAAC

**Promoter predictions for ydhB_promoter :**

**Start End Score Promoter Sequence**
 331 376 0.94 TAGGGAGTGAAATTAGTGTTGTGAAACGGTAATATTTGATGACTGGTTTC

**Promoter predictions for ydiP_promoter :**

**Start End Score Promoter Sequence**
 45 90 0.86 TGTTCTTCAGGCACCAGCTTAAAGCAGGTTATTATTTTCATGATTTCTCC
 199 244 0.85 CAACAATCTTGCAAAAAAAATGCGATCGCTAAAACAAAAGTGCAATTTGC
 299 344 0.98 TCTTGTTTAGCTCTCGCTAAACGCGGTTGTATAACCGCAATACACGCCGT
 319 364 0.92 ACGCGGTTGTATAACCGCAATACACGCCGTATTTTATCCGGCATATATTG

**Promoter predictions for ydjF_promoter :**

**Start End Score Promoter Sequence**
 19 64 0.81 CAGCGTGAAAACATGCTGAAAGTGATTGATATGCTTGAACAGTGGCAGCC
 301 346 0.94 TGTGATGAAAATCTGTGATTCAAACAGGTTATTTTGAAAGTAAACATCGG
 328 373 1.00 GTTATTTTGAAAGTAAACATCGGTTATGCGATAATCGCGCTAATGTGATG

**Promoter predictions for yeeY_promoter :**

**Start End Score Promoter Sequence**
 45 90 0.84 TCTTTGCCGGAAAAACCGCGCCTGATGGTGAACATGGTGTTAATTTAGTC

**Promoter predictions for yegW_promoter :**

**Start End Score Promoter Sequence**
 126 171 0.94 TTTTTTATCACACCAATCTGGCGGGCTATTTCAATGAATATCCGTGGTGG
 252 297 0.96 AGGTAGATGGCATCAATGGTCCGGTGGATTTTAATGTATTTAATGGCACG

**Promoter predictions for yeiL_promoter :**

**Start End Score Promoter Sequence**
 63 108 0.90 AGTTTCTGGCAAACATTCAGGCCATTAATTAATGTTTTATCAAGCGTCTG
 93 138 0.95 AATGTTTTATCAAGCGTCTGATTACCCGCTACAATAGTGATGCCTAATAA
 141 186 0.91 AAATCTATTGCCGGATGTTTCGCCGCCATCATTATAGCAATAGCATCATC
 152 197 0.82 CGGATGTTTCGCCGCCATCATTATAGCAATAGCATCATCATGACCCGGAT
 185 230 0.85 ATCATCATGACCCGGATCACAATCCAGAATAATTTTTCTCTTTTCCATTG
 232 277 0.90 TTGTTTATTTCCTCTGTTTCCAGTTGCGTTATTTTTTCTACAGCAAAGAA
 295 340 0.98 CGATGATTGAATCTTAACAACAGCGTACGTATGCTAAATATGAGAAATCT

**Promoter predictions for yfeC_promoter :**

**Start End Score Promoter Sequence**
 215 260 0.91 CGAATGTGTCAACGGCAAATTGCAACGTGTAGTTTCAATCGCTGAAAAAT
 230 275 0.95 CAAATTGCAACGTGTAGTTTCAATCGCTGAAAAATCAGGCAAATGAACAA
 308 353 0.94 TATTCATGTCACGGTTTCCTGTAAAGTGGTGTTATAAAATGAACTACTAA
 339 384 0.82 TTATAAAATGAACTACTAATAGACCCACATACATTCAGGGAATTGTTATG

**Promoter predictions for yfeD_promoter :**

**Start End Score Promoter Sequence**
 209 254 0.91 CGAATGTGTCAACGGCAAATTGCAACGTGTAGTTTCAATCGCTGAAAAAT
 224 269 0.95 CAAATTGCAACGTGTAGTTTCAATCGCTGAAAAATCAGGCAAATGAACAA
 302 347 0.94 TATTCATGTCACGGTTTCCTGTAAAGTGGTGTTATAAAATGAACTACTAA
 333 378 0.82 TTATAAAATGAACTACTAATAGACCCACATACATTCAGGGAATTGTTATG

**Promoter predictions for yfhH_promoter :**

**Start End Score Promoter Sequence**
 224 269 0.83 TAATTTTCGCTACTACATAGCCTAACAGATAGATCATCACTTTTCCGGCA
 301 346 0.97 AATTTTTAATTCATGGCAATTAGCGGCAATGGAATATAAAATTCACTCGC

**Promoter predictions for yfiE_promoter :**

**Start End Score Promoter Sequence**
 105 150 0.84 GCCAGTGAAAATGAAATGCCCGCACACAGTAACATCACAATCAAAAATCC
 309 354 0.89 CTCTGTTTTGGTAGACAGTACCCGACACGGAACATTTAAAAAAATGAATG
 341 386 0.98 CATTTAAAAAAATGAATGTTTTTATATTCTAGATTCGATATTTTCGATAG
 350 395 0.83 AAATGAATGTTTTTATATTCTAGATTCGATATTTTCGATAGGTTTGGGGT

**Promoter predictions for yfjR_promoter :**

**Start End Score Promoter Sequence**

**Promoter predictions for ygbI_promoter :**

**Start End Score Promoter Sequence**
 212 257 0.93 TCCTTGTTAATTTAAGTGATATTTTGTTTGATATTGTGAATATAAGCGCT
 220 265 0.97 AATTTAAGTGATATTTTGTTTGATATTGTGAATATAAGCGCTGGAAGATA
 234 279 0.96 TTTGTTTGATATTGTGAATATAAGCGCTGGAAGATAACGGTATGGTGATC
 245 290 0.87 TTGTGAATATAAGCGCTGGAAGATAACGGTATGGTGATCTGATTCACATA
 282 327 0.85 TCTGATTCACATAAATTAACATTGTGTGTTATTTTATGTGAACTAAGCGT

**Promoter predictions for ygeK_promoter :**

**Start End Score Promoter Sequence**
 6 51 1.00 TTAATGATTTGAATATAATCTAATTGTAATATTTTAATCATCTCATGATT
 55 100 0.98 TTTTTAATGCCTGTGGTATTTTTTTACGCAAAAATTTTATTTTTAATTAT
 73 118 0.96 TTTTTTTACGCAAAAATTTTATTTTTAATTATATTTCATTATTATTTCAT
 88 133 1.00 ATTTTATTTTTAATTATATTTCATTATTATTTCATCAATGTATTCTTTGG
 99 144 0.97 AATTATATTTCATTATTATTTCATCAATGTATTCTTTGGATTTTGCTTAT
 135 180 0.98 TGGATTTTGCTTATAATAATAATGGAAGGGATGATTATAAATGAATGCGT
 188 233 0.95 AGCATTTACACTCAGGATGAATATATGGGAAAAATTAAAATTGTAGTTTC
 224 269 0.92 AAAATTGTAGTTTCAGATCAGCAGCCGTTTATGATTGATGGGATAATTGG
 255 300 0.97 TGATTGATGGGATAATTGGATTTCTCGGACATTATCCTGATTTATATGAG
 268 313 0.97 AATTGGATTTCTCGGACATTATCCTGATTTATATGAGGTTGTTGGGGGCT
 288 333 0.89 ATCCTGATTTATATGAGGTTGTTGGGGGCTATAAAGATCTGAAGAAAGCT

**Promoter predictions for ygfI_promoter :**

**Start End Score Promoter Sequence**
 232 277 0.94 TAATATTTGTAGCCCGACGTATTCGTCGGGCTAATGCCTTTACCCGATAT
 278 323 0.90 ATATTGCCTCCTTTTTCTGTAATTTTCTGTAAAACCATGACGTAATCACC
 299 344 0.93 ATTTTCTGTAAAACCATGACGTAATCACCTATCTTTGCTCGCGGTTTCAT

**Promoter predictions for yhaJ_promoter :**

**Start End Score Promoter Sequence**
 295 340 0.91 ATAATTCTTGACCTCTCTTTACTTCCTTTTATGGTAAGGGGCTGGACGCT
 331 376 0.93 AGGGGCTGGACGCTACATTGTTAGCCAGATATTCTGCCCGGTATGTTCAA
 343 388 0.89 CTACATTGTTAGCCAGATATTCTGCCCGGTATGTTCAAATTTCCTGAATG

**Promoter predictions for yheO_promoter :**

**Start End Score Promoter Sequence**
 31 76 0.85 GAAGGTCTGAAGAACATCAAGAAAGGCGGTAAGATCAAACTGGTTATTCC
 198 243 0.80 AGCTGATGCGAAAGCCGCAGATTCTGCTAAAAAATAAGCATTAAGAACCG
 294 339 0.96 AGTGCTGGAAAGCGGAACCTCCGCTGTATTAATTTAGTTACCCGCATCAT

**Promoter predictions for yhjB_promoter :**

**Start End Score Promoter Sequence**
 29 74 0.87 GAGAAAGGTGAATCCCCTTAAACAGAGCGTATTTTTCAGCGATTCCCTTT
 138 183 0.96 ATTTTGGCTTGTGGTCGCGGAATTTCATTTTTAATTGCATGCGTACTAAC
 165 210 1.00 TTTTTAATTGCATGCGTACTAACCACCAATATAAGGAAATCCTCAAAATT
 210 255 1.00 AAATTGTTTTAAATAGTGCCCTCGACATGTAATTTCAGGCGTTTAGCTGT
 238 283 0.80 GTAATTTCAGGCGTTTAGCTGTAACTATTTATATCGGTAACTATTTTCTA
 250 295 0.87 GTTTAGCTGTAACTATTTATATCGGTAACTATTTTCTACACGTCATCTCT
 299 344 0.90 TGTAGAATGGCCACCGCCAAATGGCGAATTCTTATTTTTTCTCGCGGGTA

**Promoter predictions for yhjC_promoter :**

**Start End Score Promoter Sequence**
 33 78 0.86 GCCTGAAATTACATGTCGAGGGCACTATTTAAAACAATTTTGAGGATTTC
 56 101 0.99 ACTATTTAAAACAATTTTGAGGATTTCCTTATATTGGTGGTTAGTACGCA
 84 129 0.91 TTATATTGGTGGTTAGTACGCATGCAATTAAAAATGAAATTCCGCGACCA
 110 155 0.93 ATTAAAAATGAAATTCCGCGACCACAAGCCAAAATAACAAACGGCAAGGA
 194 239 0.97 TGTTCACTTTAAAGGGAATCGCTGAAAAATACGCTCTGTTTAAGGGGATT
 304 349 0.87 ACAACATTGTGAAACCCGGCATTAGATGTTAGAAAAAACAATAAACAATG

**Promoter predictions for yiaG_promoter :**

**Start End Score Promoter Sequence**
 88 133 0.90 GGAGAAAATGAAACAAGGTTAATTTGTTGTTTCATTGTTAAAAAATGATA
 197 242 0.95 AATCTTTACAAAACAGGAGTGGTAAGAGATATATCGGCATTTATCAGATC

**Promoter predictions for yiaU_promoter :**

**Start End Score Promoter Sequence**
 57 102 0.96 TTTTTTTCACTTTGCGACAGAATAAAACCTAACGTGGTCTGACGAACATA
 149 194 0.80 ATTTTCATTATAACCGCGATAAGGCGATTCATTATAAGCCGCACCAGCAC
 203 248 0.84 GGATAATTCAGAAGCAGTTGCGCTTGCCATAAAAGGCAACGCAAATAACG
 265 310 0.98 GATTAATTAACATGTCCGGTATTCCATTTTAAAATAAGATAAAAAAGAGT
 289 334 1.00 CATTTTAAAATAAGATAAAAAAGAGTCGGCATAATAATGCTTACAATTTC
 354 399 0.89 ATATTTATATAGTAATATATAAAATTATATATAATTGGGCTGTTGCGATG

**Promoter predictions for yidL_promoter :**

**Start End Score Promoter Sequence**

**Promoter predictions for yidP_promoter :**

**Start End Score Promoter Sequence**
 2 47 0.97 GGGTTTTGCAGCAAGATGGCAAGACCCACCACAATCCCGGCGAAGGGAAA
 311 356 0.97 ACATCTTTAAAAAAAAGATGTTTTTTCAATCGATTAAGCAGAACTTGTGT

**Promoter predictions for yieP_promoter :**

**Start End Score Promoter Sequence**
 72 117 0.83 TTTGCTTTTTCTCAGCGGCGCGGGGTGTGCATAATACGCTTTCCCGCTAC
 250 295 0.94 AGGAAATTTAAAATAATTTTCTGACCGCGCATTTTTTCAGCAAAAGCCCC

**Promoter predictions for yihL_promoter :**

**Start End Score Promoter Sequence**
 333 378 0.82 GTTAATTGCTTTCTTTTTTGGCGTAAGCGTAAGATGCTTCATCTGGTTTA

**Promoter predictions for yihW_promoter :**

**Start End Score Promoter Sequence**
 34 79 0.88 AACGGGCGTGAAAGAGATGGCCAGTGCGCTAAAACAGGCACAAACGCTCA

**Promoter predictions for yiiF_promoter :**

**Start End Score Promoter Sequence**
 152 197 0.83 AGGAATGACAAAAATTTTTCATTCTTGAATATAAAAAACAGATGCCCTTA
 171 216 0.94 CATTCTTGAATATAAAAAACAGATGCCCTTATTCTGGTATTAATACAAGG
 228 273 0.91 ACTTGAACTTATAATAACTGCAACTGTTACATCATATCTGGAAAACGCCT
 259 304 0.97 TCATATCTGGAAAACGCCTCGCAAAAATAAAAAATGATGCGTAAATGAGC
 330 375 0.93 GGTACTTTTCTCTCCATAGATTCAAAAATGATATTGGCGAGATATTTATG

**Promoter predictions for yijO_promoter :**

**Start End Score Promoter Sequence**
 26 71 0.96 CAGATTTGGCGGGCTTATCTTACGACGGTTACGATCCAACCCGTTCAGTG
 200 245 0.91 GCTATTTGCCGGATGCGACGCTTGACGCGTCTTATCCGGCCTACAGATCG

**Promoter predictions for yjdC_promoter :**

**Start End Score Promoter Sequence**
 12 57 0.93 ACTTTACACAAATCAAAACGGTAGATGAGTTAAATCAGGCGCTCGTTGAA

**Promoter predictions for yjhI_promoter :**

**Start End Score Promoter Sequence**
 74 119 0.88 GTTTTTACCTCATTCAATAAAGTGATAAGTAAGTTCATTCGAGAGGGATT
 104 149 0.86 AAGTTCATTCGAGAGGGATTTCAAGCAAAAATAATCAATGGCACCCAATA
 212 257 0.92 ACGATGTTGCAACAACACGCCATCTACTTTTTATTCTCATTCACTAAATG
 276 321 0.97 TCATTATTCAAAGTGTGTACAAGATCACATTTAATCACATCATTACGGTT
 328 373 0.81 GCATGCTGAACAAAGCATATTTTCCACTATGTAATGCCGATACCATTTAT
 344 389 0.87 ATATTTTCCACTATGTAATGCCGATACCATTTATTCCATGAGCAAGGAGG

**Promoter predictions for yjiR_promoter :**

**Start End Score Promoter Sequence**
 8 53 1.00 CTTAATTTAAAAACATAAAAACGTTTTATTATATATAAATCTATATCCCC
 263 308 0.85 CTTCTGGCAGTTCACAATACAGATTCAAATATTACTTTTATTTAACATAC
 297 342 0.95 CTTTTATTTAACATACAGACAGGTTAAAACAGCATCTGCATCCGGTTTTT

**Promoter predictions for yneJ_promoter :**

**Start End Score Promoter Sequence**
 47 92 0.98 TGGTTGATTGGTTTGCCCATTTCGCGGGTGATCATTTGCGCCATTTCTTC
 240 285 0.84 AAAGTTGTTCACCCGTGGCAGGATTTATCGAAATTGCATGAGTTGCCGGA
 336 381 0.83 ACGCAGATTTACTCTTGCTTTAAAATGAATAATATTAAGCCACTTATTCA

**Promoter predictions for yneL_promoter :**

**Start End Score Promoter Sequence**
 146 191 0.95 CCTTGGTTTATATTGGTGCGCATAATGTATATTATGTTAAATCATGTCTG

**Promoter predictions for ynfL_promoter :**

**Start End Score Promoter Sequence**
 336 381 0.85 ATAGGGTAGGAAAATCGAATTGTTCTGTCTAATATATTAATAATCTCAAA
 352 397 0.91 GAATTGTTCTGTCTAATATATTAATAATCTCAAATAAGATGTTTTAAATA

**Promoter predictions for ypdC_promoter :**

**Start End Score Promoter Sequence**
 195 240 0.91 CCGAATTTTGCAGCAAACTGCCGCCGTCGCATTTTTTCCGCTGCCATCGC
 323 368 0.99 CTGGATTTTGAAGTGCCGGTCAGCCGCAGCAAAGTGAAAGAATTTCGCCA

**Promoter predictions for yphH_promoter :**

**Start End Score Promoter Sequence**
 335 380 0.93 TATTGAACAATCTGGCAATGTTTTCGCGGAATAATCACGCAATTAACTAA

**Promoter predictions for yqeH_promoter :**

**Start End Score Promoter Sequence**
 74 119 0.82 GAGATCTGAACATCTTAAATAAGACTATTTAAGATGCATAACTTAGATTC
 83 128 0.81 ACATCTTAAATAAGACTATTTAAGATGCATAACTTAGATTCGCAAGATAT
 188 233 0.81 TTATCTGGACCGCTCTCAACCATGCAAGATTAAATCAGTGAAACATAATC
 234 279 0.86 AATCATATTTGATAACGAGAAATGCATTTTTAAATGCCTTACTCAGAATT
 254 299 0.83 AATGCATTTTTAAATGCCTTACTCAGAATTATAGCAAATACAGATTAATC
 316 361 0.83 GGCAAAATTACAAAATCATGGTGTATTGACATCATCAATAACCAATGAAA
 335 380 0.96 GGTGTATTGACATCATCAATAACCAATGAAATTATGCAATTATATACGGA

**Promoter predictions for yqeI_promoter :**

**Start End Score Promoter Sequence**
 57 102 0.82 TCCAACATTGATACTCAACGACCAGCCAGAATCATACTCTGTTTATACGG
 222 267 0.96 AACAATAGTGGCATAAATGTTAACCATGTTAATTTACGTAAAGTTTTACG
 266 311 0.85 TTTACGTTGCAACATTAAAGCCTCATTTCAATCATCATGATAAATATAAA
 289 334 0.90 CATTTCAATCATCATGATAAATATAAAATTAATATATATTTATGCCGTAA

**Promoter predictions for ytfH_promoter :**

**Start End Score Promoter Sequence**
 278 323 0.97 CCAAGTTGGCCAGTGGCACCAGTAATAGCGATCATGGGAAGTCTCCATCG
 335 380 0.94 TTGTGTTTAAATACGCTAACACCTAAACTTACTTTTAGTAAGTACGTACA

**PePPER: a webserver for prediction of prokaryote promoter elements and regulons**

Name ID Position Strand Score Sequence

P_0001 yfeD_promoter 191 - 10.656689379 TTGACACATTCGGGCGGAATTCATATGAT

P_0002 yfeD_promoter 52 + 8.32228499658 TTTACGTACCAAGTTTGCTGGGTGCAAAAT

P_0003 ybdO_promoter 283 - 10.2780879436 TTAACTCAGGATTAAGTTTTTCTAAAAT

P_0004 yneJ_promoter 342 + 10.3649024511 TTTACTCTTGCTTTAAAATGAATAATAT

P_0005 yiaU_promoter 270 + 9.80366369168 TTAACATGTCCGGTATTCCATTTTAAAAT

P_0006 ycaN_prmoter 332 - 9.33797436477 TTTACAGGATTATGCCGCCTGCGTAAAGT

P_0007 yfeC_promoter 197 - 10.656689379 TTGACACATTCGGGCGGAATTCATATGAT

P_0008 yfeC_promoter 58 + 8.32228499658 TTTACGTACCAAGTTTGCTGGGTGCAAAAT

P_0009 yieP_promoter 101 - 11.1311136309 TTGACTCTGTAGCGGGAAAGCGTATTAT

P_0010 ybeF_promoter 359 + 10.4079128701 TTTAAATATTATTTTTCCATGAATAAAAT

Name ID Position Strand Score Sequence

P_0001 ycaN_prmoter 332 - 9.33797436477 TTTACAGGATTATGCCGCCTGCGTAAAGT

P_0002 yfeD_promoter 191 - 10.656689379 TTGACACATTCGGGCGGAATTCATATGAT

P_0003 yfeD_promoter 52 + 8.32228499658 TTTACGTACCAAGTTTGCTGGGTGCAAAAT

P_0004 ybeF_promoter 359 + 10.4079128701 TTTAAATATTATTTTTCCATGAATAAAAT

P_0005 ybdO_promoter 283 - 10.2780879436 TTAACTCAGGATTAAGTTTTTCTAAAAT

P_0006 yieP_promoter 101 - 11.1311136309 TTGACTCTGTAGCGGGAAAGCGTATTAT

P_0007 yfeC_promoter 197 - 10.656689379 TTGACACATTCGGGCGGAATTCATATGAT

P_0008 yfeC_promoter 58 + 8.32228499658 TTTACGTACCAAGTTTGCTGGGTGCAAAAT

P_0009 yneJ_promoter 342 + 10.3649024511 TTTACTCTTGCTTTAAAATGAATAATAT

P_0010 yiaU_promoter 270 + 9.80366369168 TTAACATGTCCGGTATTCCATTTTAAAAT
