# Supplementary material for: Escherichia coli transcription factors of unknown function: sequence features and possible evolutionary relationships
Source: PeerJ. 2022 Jul 20;10:e13772. doi: 10.7717/peerj.13772 (PMC9308461; doi:10.7717/peerj.13772)
Supplement: Supplemental Information 16 — The TF analyzed is in bold. [file peerj-10-13772-s016.docx]

Supplementary references and experimental conditions for Figure 6 expression data. The expression data for each condition is given for each TF indicated in bold. Representative experiments are shown that provide either strong expression or repression; or is relevant to the STRING prediction. The reference and data were extracted from: https://genexpdb.okstate.edu/databases/genexpdb/

[1] Massé E, Vanderpool CK, Gottesman S. Effect of RyhB small RNA on global iron use in *Escherichia coli*. J Bacteriol. 2005 Oct;187(20):6962-71. doi: 10.1128/JB.187.20.6962-6971.2005. Description: Effect of small RNA RyhB on global iron use in *Escherichia coli*. FeSO_4_, *fur; ryhB* overexpression. Result observed: No change in the control condition (first data). Repression with *fur* overexpression, second data and mild repression with FeSO_4_ present, third data. **yahB:** same data analysis.

[2] Riehle MM, Bennett AF, Lenski RE, Long AD. Evolutionary changes in heat-inducible gene expression in lines of *Escherichia coli* adapted to high temperature. Physiol Genomics. 2003 Jun 24;14(1):47-58. doi: 10.1152/physiolgenomics.00034.2002. Description: High temperature adapted coli expression. Evolution in chemostat at 41.5 degrees C • *E. coli* B strain Bc251 (REL606) Ara-. Results shown: increased expression in high temperature evolved line 42-1 vs ancestor, first data. Third data, increased expression in high temperature evolved line 42-3 vs ancestor. **yhaJ:**, same analysis. **yahB:** same analysis. **yafC:** same analysis. **yieP**: same analysis, activated in evolved lines 42-1 and 42-2. **yihL:** activated expression in evolved lines 42-2 and 42-3. **ybeF:** strongly repressed in evolved strains 42-1, 42-2 and 42-3.

[3] Ito A, May T, Taniuchi A, Kawata K, Okabe S. Localized expression profiles of *rpoS* in *Escherichia coli* biofilms. Biotechnol Bioeng. 2009 Aug 1;103(5):975-83. doi: 10.1002/bit.22305. Description: Gene expression of *E. coli* MG1655 pOX38Km at the outside and inside of biofilms. Biofilm, planktonic, exponential phase. **yneJ:** Results shown: First data, repression in biofilm outside/biofilm inside. Second data shown, repression in planktonic exponential phase/planktonic stationary phase. Third data shown, increased activation biofilm inside/planktonic exponential phase. Fourth data shown: mild increased expression in biofilm outside/planktonic exponential phase. **yfiE:** Results shown: First data, repression in biofilm outside/biofilm inside. Second data shown, mild repression in planktonic exponential phase/planktonic stationary phase. Third data shown, increased activation biofilm inside/planktonic exponential phase. Fourth data shown: repression in biofilm outside/planktonic exponential phase. **yqeI:** activation during biofilm formation and *rpoS* mutant in exponential and stationary growth. **yijO:** higher expression on cells outside of the biofilm than the cells inside the biofilm. In concordance with the observed results in reference [10]. **yagA:** repressed in planktonic exponential phase and repressed in biofilm outside. **ybaQ:** repressed in planktonic exponential phase and low expression in biofilm inside.

[4] Worden CR, Kovac WK, Dorn LA, Sandrin TR. Environmental pH affects transcriptional responses to cadmium toxicity in *Escherichia coli* K-12 (MG1655). FEMS Microbiol Lett. 2009 Apr;293(1):58-64. doi: 10.1111/j.1574-6968.2009.01508.x. Description: Effect of pH on Cadmium Toxicity in *Escherichia coli*. 1 µg/ml CdCl_2_ • M9 glucose. **yfiE:** Results shown: First data, strong activation, 5min 1 µg/mL Cd pH7/5 min 1µg/mL Cd pH 5. Second data, no change in expression, 5 min no Cd pH 7/ 5 min no Cd pH 5. Third data, mild increase in expression, 15 min 1 µg/mL Cd pH7/5 min 1 µg/mL Cd pH5. Same pattern observed in: Helbig K, Grosse C, Nies DH. Cadmium toxicity in glutathione mutants of *Escherichia coli*. J Bacteriol. 2008 Aug;190(15):5439-54. doi: 10.1128/JB.00272-08. **ydjF:** activation after 5 min 1 µg/mL Cd pH 7/ 1 µg/mL Cd pH 5. Strong repression after 15 min 1 µg/mL Cd pH 7/ 5 min 1µg/mL Cd pH 5. **ydcR:** repressed with 5 min 1 µg/mL Cd pH7/5 min 1 µg/mL Cd pH 5, strong expression with no Cd but pH difference.

[5] de Pace F, Nakazato G, Pacheco A, de Paiva JB, Sperandio V, da Silveira WD. The type VI secretion system plays a role in type 1 fimbria expression and pathogenesis of an avian pathogenic *Escherichia coli* strain. Infect Immun. 2010 Dec;78(12):4990-8. doi: 10.1128/IAI.00531-10. Description: Expression data from an Avian pathogenic *Escherichia coli* strain. *hcp*, *clpV*, *icmF*. **yfiE:** Results shown: First data, activation in *clpV* deleted mutant/ WT. Second data, activation in *hcp* deleted mutant/ WT. Third data, activation in *icmF* deleted mutant/ WT.

[6] Kao KC, Tran LM, Liao JC. A global regulatory role of gluconeogenic genes in *Escherichia coli* revealed by transcriptome network analysis. J Biol Chem. 2005 Oct 28;280(43):36079-87. doi: 10.1074/jbc.M508202200. Description: Glucose to acetate transition. Time series • glucose-acetate shift • ppsA. Data points shown here indicate the transition of expression of cells shifted from glucose to acetate at different times and in comparison, with *ppsA* mutant, coding for the enzyme that catalyzes the phosphorylation of pyruvate to phosphoenolpyruvate. **ynfL*:*** Same data analysis. **yeeY:** same analysis. **yhaJ:** same analysis. **ydhB:** same analysis. **yiaU:** same analysis. **yjhJ:** activation upon glucose to acetate shift.

[7] Ernst J, Beg QK, Kay KA, Balázsi G, Oltvai ZN, Bar-Joseph Z. A semi-supervised method for predicting transcription factor-gene interactions in *Escherichia coli*. PLoS Comput Biol. 2008 Mar 28;4(3):e1000044. doi: 10.1371/journal.pcbi.1000044. Description: Dynamics of *E. coli* Aerobic to Anaerobic Switch Response. Time series • aerobic to anaerobic shift. Expression reduction during aerobic- anaerobic shift. **ygfI:** expression during aerobic to anaerobic shift. **ybiH:** expression during aerobic to anaerobic shift.

[8] Berney M, Weilenmann HU, Egli T. Gene expression of *Escherichia coli* in continuous culture during adaptation to artificial sunlight. Environ Microbiol. 2006 Sep;8(9):1635-47. doi: 10.1111/j.1462-2920.2006.01057.x. Description: Gene expression of *E. coli* in continuous culture during adaptation to artificial sunlight. UV light • evolution in chemostat; stress. Data shown: first data, UV irradiated 50 h/ not irradiated. Second data: UV irradiated 1 h/ not irradiated. Third data, UV irradiated 1 h / not irradiated (Dye swap). **ydcR:** strongly expressed in UV irradiated 50 h, mild expression after 1 h UV irradiation. **ydaW:** strong expression in UV irradiated for 50 h. Mild expression in UV irradiated 1 h.

[9] Constantinidou C, Hobman JL, Griffiths L, Patel MD, Penn CW, Cole JA, Overton TW. A reassessment of the FNR regulon and transcriptomic analysis of the effects of nitrate, nitrite, NarXL, and NarQP as *Escherichia coli* K12 adapts from aerobic to anaerobic growth. J Biol Chem. 2006 Feb 24;281(8):4802-15. doi: 10.1074/jbc.M512312200. Description: A reassesment of the FNR regulon and the effects of nitrate,nitrite, narXL and narQP aerobic; anaerobic; NO_2_; NO_3_ • *fnr*; *narXL*; *narXLP*. Data shows the effect of deleting either *fnr*; *narXL*; *narXLP* in comparison with the Wt. Shows repression in the deletions, the shift to anaerobiosis and in the presence of NO_2_; NO_3_.

[10] Ito A, May T, Kawata K, Okabe S. Significance of *rpoS* during maturation of *Escherichia coli* biofilms. Biotechnol Bioeng. 2008 Apr 15;99(6):1462-71. doi: 10.1002/bit.21695. Gene expression of *E. coli* MG1655 wild type and *rpoS* knock-out strains’ biofilm; stationary phase • *rpoS*. Data: first data point, *rpoS* mutant biofilm/ Wt biofilm. Activation, second data point, Wt biofilm/ Wt exponential growth. Third data point, *rpoS* mutant exponential growth /Wt exponential growth. Fourth data point: Wt stationaryphase / Wt exponential. Fifth data point, *rpoS* mutant stationary phase/ Wt stationary phase. **yheO:** highly expressed in *rpoS* mutant strain biofilm formation and repressed in the wt strain.

[11] Faith JJ, Hayete B, Thaden JT, Mogno I, Wierzbowski J, Cottarel G, Kasif S, Collins JJ, Gardner TS. Large-scale mapping and validation of *Escherichia coli* transcriptional regulation from a compendium of expression profiles. PLoS Biol. 2007 Jan;5(1):e8. doi: 10.1371/journal.pbio.0050008. Description: arge-Scale Mapping and Validation of E. coli Transcriptional Regulation from a Compendium of Expression Profiles. Ampicillin; norfloxacin; arabinose; luciferase; stationary phase • *luc; dinI; dinP; lexA; lon; luc; recA; ruvA; sulA; umuD; uvrA; dnaA; gyrA; gyrI; minD; murI; rstB; uspA; menB; dnaN; sbcB; hscA; minE; emrR; holD; ihf; minE; nrdA; nrdB; hlpA; holD; hscA; ruvC; folA; menC; galF; nupC; gcvR; gyrI; minD; murI; rimI; rs*. For **ydhB**, the expression profile where activation is observed is in the presence of norfloxacin along with other mutations, in the *recA* mutant and repressed in metabolic shift conditions.

[12] Cardinale CJ, Washburn RS, Tadigotla VR, Brown LM, Gottesman ME, Nudler E. Termination factor Rho and its cofactors NusA and NusG silence foreign DNA in *E. coli*. Science. 2008 May 16;320(5878):935-8. doi: 10.1126/science.1152763. Description: Genome-wide analysis of transcriptional termination in *E. coli*. Bicyclomycin • nusA; nusG; O157:H7 strain EDL933; MDS42 reduced genome strain. **yiaU:** activation in MG1655 strain with 25 and 100 µg bicyclomycin, strong activation in O157:H7 strain with 100 µg bicyclomycin. Also, activation in genome reduced strain MDS42 with 100 µg bicyclomycin. Activated in the same strain in knockout of *nusG*. **yidP:** expression with 100 µg bicyclomycin and in *nusG* mutant. **yiiF:** expression in the presences of bicyclomycin in both the wt MG1655 strain and the *nusG* mutant, the later being the highest expression. **ygeK pseudogene:** surprisingly this gene is expressed strongly in the presence of bicyclomycin in all conditions with controls strains but not in the *nusG* mutant. **ybeF:** strongly expressed in 100 µg of bicyclomycin in MG1655, O157:H7and MDS42 strains. **yijO:** higher expression on *nusG* mutant strain, also expression is observed with bicyclomycin. **YqeH:** expression in O157:H7 and MDS42 strains with bicylcomycin. **yaiV:** expression found in 25 µg and 100 µg bicyclomicin in the MG1655 strain and 100 µg bicyclomicin in strain I157:H7 strain. Interestingly, in the genome-reduced strain MDS42 is not expressed.

[13] Sangurdekar DP, Srienc F, Khodursky AB. A classification based framework for quantitative description of large-scale microarray data. Genome Biol. 2006;7(4):R32. doi: 10.1186/gb-2006-7-4-r32. Description: Anaerobic growth in M9 + glucose, genomic DNA reference. Growth rate comparison • anaerobic. Strong activation during anaerobic growth in M9 and glucose. **ygfL:** analysis of expression during stationary phase, repressed.

[14] Brynildsen MP, Liao JC. An integrated network approach identifies the isobutanol response network of *Escherichia coli*. Mol Syst Biol. 2009;5:277. doi: 10.1038/msb.2009.34. Description: Identification of isobutanol response network of *E. coli*. isobutanol, n-butanol, ethanol • aerobic • *fur, phoB, arcA, ubiE, ihfA*. Activation in the presence of isobutanol in different genetic backgrounds.

[15] Jozefczuk S, Klie S, Catchpole G, Szymanski J, Cuadros-Inostroza A, Steinhauser D, Selbig J, Willmitzer L. Metabolomic and transcriptomic stress response of *Escherichia coli*. Mol Syst Biol. 2010 May 11;6:364. doi: 10.1038/msb.2010.18. Description: Time resolved response of *E. coli* to 4 different stress conditions time series • 200 µg/ml H_2_O_2_, 0.15% lactose plus 0.05% glucose, 45°C, 16°C. For **yhjC** the gene is repressed in cold stress and activated in heat stress. Consistent with reference [2]. **yfeC:** repressed in oxidative and heat stress. Activated in cold stress and glucose-lactose shift. Not detected in dataset of reference [2]. **yfeD:** Activated in cold stress and repressed in heat stress and oxidative stress, also repressed in glucose-lactose shift. Consistent with reference [2]. **yjhJ:** strongly activated in glucose-lactose shift. **ygbI:** mild activation in glucose-lactose shift and heat shock. **ydjF:** activation with heat stress, and glucose-lactose shift, repression with oxidative stress. **yihW:** activated in heat stress and glucose-lactose shift. **yieP:** activated in cold shock and repressed in heat shock. Mild activation in glucose-lactose shift. **yegW:** mild activation with cold shock and repression with glucose-lactose shift. **yidP:** expression in oxidative stress and glucose-lactose shift. **yihL:** repression during heat and oxidative stress. Mild activation during glucose-lactose shift. **yahA:** repressed in cold stress, activated in heat stress, and regulated in glucose-lactose shift. **yiiF:** repressed in the conditions tested. **ybaQ:** expression in glucose-lactose shift and heat stress. **yiaG:** repression in cold stress, activation in heat stress and glucose-lactose shift. **yjdC:** Expressed in heat stress. Mild repression in cold stress. Mild activation in glucose-lactose shift. **yagI:** strong repression in glucose-lactose shift. Also, in cold and oxidative stress. **yddM:** expression in cold and heat stress, repressed in oxidative stress and glucose-lactose shift. **ytfH:** strong expression is found in heat stress; also, mild expression is found in oxidative stress and glucose-lactose shift. **yhjB:** expression observed during oxdative stress, limited expression observed in glucose-lactose shift. **yfhH:** clear regulation by temperature stress, repressed in cold and activated in heat stress. **ygiP:**  express in heat stress and repressed in cold and oxidative stress. Also, in glucose-lactose shift. **yneL:** strong expression in heat stress. Repressed in cold stress and oxidative stress.

[16] Courcelle J, Khodursky A, Peter B, Brown PO, Hanawalt PC. Comparative gene expression profiles following UV exposure in wild-type and SOS-deficient *Escherichia coli*. Genetics. 2001 May;158(1):41-64. doi: 10.1093/genetics/158.1.41. Description: UV Exposure in Wild-Type and SOS-Deficient *E. coli*. Time Series • UV light • stress; SOS response • *lexA*. **yqeI:** repression upon UV irradiation.

[17] Tehranchi AK, Blankschien MD, Zhang Y, Halliday JA, Srivatsan A, Peng J, Herman C, Wang JD. The transcription factor DksA prevents conflicts between DNA replication and transcription machinery. Cell. 2010 May 14;141(4):595-605. doi: 10.1016/j.cell.2010.03.036. Description: Expression profiling of *E. coli* K-12 wild-type vs. delta *dksA* cells upon amino acid starvation. Serine hydroxamate (SHX) • DksA. **yjiR:** repression in Wt and DksA deficient cells, activated in cells treated with SHX either wt or mutant strain.

[18] Bernstein JA, Khodursky AB, Lin PH, Lin-Chao S, Cohen SN. Global analysis of mRNA decay and abundance in *Escherichia coli* at single-gene resolution using two-color fluorescent DNA microarrays. Proc Natl Acad Sci U S A. 2002 Jul 23;99(15):9697-702. doi: 10.1073/pnas.112318199. Description: Global analysis of mRNA decay and abundance in *Escherichia coli*. mRNA decay • LB; M9. **yheO:** repressed after exposure to rifampicin, expressed in wt cells in M9 glucose.

[19] Domka J, Lee J, Bansal T, Wood TK. Temporal gene-expression in *Escherichia coli* K-12 biofilms. Environ Microbiol. 2007 Feb;9(2):332-46. doi: 10.1111/j.1462-2920.2006.01143.x. Description: Temporal Biofilm Expression of *E. coli* K-12 after 4, 7, 15, and 24 hr. Time Series • biofilm; suspension. **yfjR:** highly expressed after different times of biofilm formation. **ydiP:** repressed in the wt after 15 h of suspension culture and highly expressed in wt biofilm after 15 h of culturing. **ydaW:** Expression in biofilm after 15 h and 24 h culture.

[20] Liu M, Durfee T, Cabrera JE, Zhao K, Jin DJ, Blattner FR. Global transcriptional programs reveal a carbon source foraging strategy by *Escherichia coli*. J Biol Chem. 2005 Apr 22;280(16):15921-7. doi: 10.1074/jbc.M414050200. Description: carbon utilization in *E. coli*. Glucose; glycerol; succinate; L-alanine; acetate; L-proline; carbon source. **yahA:** repressed in glycerol, succinate, alanine, acetate and proline as carbon sources.

[21] Oberto J, Nabti S, Jooste V, Mignot H, Rouviere-Yaniv J. The HU regulon is composed of genes responding to anaerobiosis, acid stress, high osmolarity and SOS induction. PLoS One. 2009;4(2):e4367. doi: 10.1371/journal.pone.0004367. Description: Identification of the *Escherichia coli* HU regulon. Exponential, transition, stationary phases; LB, M9 glucose, glycerol • *hupA, hupB, hupAB, rpoS*. **ygeK:** activation in Wt stationary phase, in *hupA* mutant, strongly expressed in *hupB* mutant and in *rpoS* mutant mild expression is observed. Other conditions showed repression or no change, such as in the *hubB* mutant vs Wt LB transition. Perhaps part of the HU regulon.

[22] Hyduke DR, Jarboe LR, Tran LM, Chou KJ, Liao JC. Integrated network analysis identifies nitric oxide response networks and dihydroxyacid dehydratase as a crucial target in *Escherichia coli*. Proc Natl Acad Sci U S A. 2007 May 15;104(20):8484-9. doi: 10.1073/pnas.0610888104. Description: Identification of NO Response Network in *E. coli*. serine hydroxamate; DeaNO; methionine • *argR; arcA; nsrR; iscR; hmpA; norR; metJ*. **yphH:** *argR* mutant showed decreased expression. *metJ* mutant showed activation. *nsrR*, showed activation. May be involved in the nitric oxide regulatory network.

[23] Blanchard JL, Wholey WY, Conlon EM, Pomposiello PJ. Rapid changes in gene expression dynamics in response to superoxide reveal SoxRS-dependent and independent transcriptional networks. PLoS One. 2007 Nov 14;2(11):e1186. doi: 10.1371/journal.pone.0001186. Description: Expression data from a paraquat time course experiment in wild type and SoxR deficient strains. Time Series • paraquat • *soxR*. **ybdO:** clearly its expression is SoxR-dependent and time of exposure to paraquat. **ybaO:** clearly its expressed in a SoxR-dependent and time of exposure to paraquat.

[24] Traxler MF, Chang DE, Conway T. Guanosine 3',5'-bispyrophosphate coordinates global gene expression during glucose-lactose diauxie in *Escherichia coli*. Proc Natl Acad Sci U S A. 2006 Feb 14;103(7):2374-9. doi: 10.1073/pnas.0510995103. Description: Time series analysis of glucose-lactose diauxie: involvement of stringent response. Time Series • diauxie; stationary phase • *crp relA rpoS*. **yeiL:** repressed in a wide range of conditions involving stringent response, including the *rpoS* mutant. Repression partially alleviated in a *relA* nutant.

[25] Ito A, Taniuchi A, May T, Kawata K, Okabe S. Increased antibiotic resistance of *Escherichia coli* in mature biofilms. Appl Environ Microbiol. 2009 Jun;75(12):4093-100. doi: 10.1128/AEM.02949-080. Description: Gene expression of *E. coli* MG1655 at attachment, colony formation and maturation during biofilm formation. Biofilm, planktonic, exponential phase, stationary phase **yidL:** repression in biofilm maturation stage and expression in attachment stage and planktonic exponential phase. May be involved in the initiation and maturation of the biofilm formation. **ypdC:** repressed in attachment stage and expressed in planktonic exponential phase. **yaiV:** repressed in attachment stage and expressed during biofilm formation and planktonic exponential phase.

[26] Moen B, Janbu AO, Langsrud S, Langsrud O, Hobman JL, Constantinidou C, Kohler A, Rudi K. Global responses of *Escherichia coli* to adverse conditions determined by microarrays and FT-IR spectroscopy.Can J Microbiol. 2009 Jun;55(6):714-28. doi: 10.1139/w09-016. Description: Overlapping and unique responses of *E. coli* to adverse conditions. Expression profiling by array.  **yagA:** repressed in 4.5% NaCL in TSB. Expressed in 15% glycerol, NaOH (pH 9.6), Heat (46°C), 5% ethanol and Cold (15°C). Cold stress correlates with the reported in [15], also in [15] repression in glucose-lactose shift is observed.

[27] Traxler MF, Summers SM, Nguyen HT, Zacharia VM, Hightower GA, Smith JT, Conway T. The global, ppGpp-mediated stringent response to amino acid starvation in *Escherichia coli*. Mol Microbiol. 2008 Jun;68(5):1128-48. doi: 10.1111/j.1365-2958.2008.06229.x. Description: *E. coli* Isoleucine starvation and stringent response network. Time series • amino acid starvation • *relA, spoT, crp, dksA, lrp, rpoS*. **yiaG:** Expression in isoleucine starved Wt, *crp* and *lrp* strains. Repressed in *rpoS* isoleucine starved. **ybcM:** isoleucine starved activation in the Wt and the mutant backgrounds tested.

[28] Helbig K, Grosse C, Nies DH. Cadmium toxicity in glutathione mutants of *Escherichia coli*. J Bacteriol. 2008 Aug;190(15):5439-54. doi: 10.1128/JB.00272-08. Description: response of *Escherichia coli* and glutathion mutants to Cadmium. Cadmium • *gshA; gshB*. **ycfQ:** expression in the wt and glutathione mutants in the presence of cadmium.

[29] Sangurdekar DP, Srienc F, Khodursky AB. A classification based framework for quantitative description of large-scale microarray data. Genome Biol. 2006;7(4):R32. doi: 10.1186/gb-2006-7-4-r32. Description: Anaerobic growth in M9 + glucose, genomic DNA reference. Growth rate comparison • anaerobic. **ycjW:** strong expression at OD 0.2, 0.35, 0.6 and 0.9 anaerobic growth in M9 + glucose, expression is reduced in stationary phase in the same condition.

Many of the transcription factors that responded to metabolic shifts also showed expression patterns related to the presence of amino acids, such as the reported by Khodursky *et al*. (2000). Khodursky AB, Peter BJ, Cozzarelli NR, Botstein D, Brown PO, Yanofsky C. DNA microarray analysis of gene expression in response to physiological and genetic changes that affect tryptophan metabolism in *Escherichia coli*. Proc Natl Acad Sci U S A. 2000 Oct 24;97(22):12170-5. doi: 10.1073/pnas.220414297.Description: Tryptophan metabolism response in *E. coli* Time Series • L-tryptophan; indole acrylate • *trpR; trpE; trpD; trpC; trpB; trpA; tnaA*.
